# Supplementary material for: Hexanematic crossover in epithelial monolayers depends on cell adhesion and cell density
Source: Nat Commun. 2023 Sep 16;14:5762. doi: 10.1038/s41467-023-41449-6 (PMC10505199; doi:10.1038/s41467-023-41449-6)
Supplement: Supplementary file 1 — Supplementary Information [file 41467_2023_41449_MOESM1_ESM.pdf]

# Supplementary Information

## Hexanematic crossover in epithelial monolayers depends on cell adhesion and cell density

Julia Eckert[‡1], Benoît Ladoux[2], René-Marc Mège[2], Luca Giomi[3] and Thomas Schmidt[1]

[1]Physics of Life Processes, Leiden Institute of Physics, Universiteit Leiden, 2333 CC Leiden, The Netherlands.
[2]Université Paris Cité, CNRS, Institut Jacques Monod, F-75013 Paris, France.
[3]Instituut-Lorentz, Leiden Institute of Physics, Universiteit Leiden, P.O. Box 9506, 2300 RA Leiden, The Netherlands.
[‡]Current address: Centre for Cell Biology of Chronic Disease, Institute for Molecular Bioscience, The University of Queensland, St. Lucia, Brisbane, Queensland, Australia 4072.

**Table S1** Number of experiments, samples, imaged monolayers, and analyzed MDCK-II WT and E-cad KO cells on non-coated glass, 25 kPa, and 49 kPa. Source data are provided as Source Data file.

| | | experiments | samples | images | total numner of cells |
|---|---|---|---|---|---|
| WT | glass | 3 | 16 | 226 | 11987 |
| | 25 kPa | 5 | 8 | 95 | 9391 |
| | 49 kPa | 3 | 6 | 75 | 4485 |
| E-cad KO | glass | 3 | 18 | 216 | 6941 |
| | 25 kPa | 3 | 7 | 81 | 5412 |
| | 49 kPa | 3 | 11 | 144 | 8348 |

**Table S2** Comparison of the differences between the hexatic and nematic shape functions, $\langle|\gamma_6|\rangle - \langle|\gamma_2|\rangle$, at different monolayer densities for MDCK-II WT and E-cad KO cells on glass. Cells were classified into groups D1-D6 according to their cell-cell distance. Statistical test: Dunn's test of multiple comparisons after a significant Kruskal–Wallis test. p-value $> 0.05$, difference is not significantly different. Source data are provided as Source Data file.

| WT | $\langle|\gamma_6|\rangle - \langle|\gamma_2|\rangle$ | | | |
|---|---|---|---|---|
| p-value | D1 | D2 | D3 | D4 |
| D2 | 0.08 | | | |
| D3 | 0.020 | 0.23 | | |
| D4 | 0.041 | 0.31 | 0.44 | |
| D5 | 0.0006 | 0.0036 | 0.007 | 0.007 |

| E-cad KO | $\langle|\gamma_6|\rangle - \langle|\gamma_2|\rangle$ | | |
|---|---|---|---|
| p-value | D3 | D4 | D5 |
| D4 | 0.37 | | |
| D5 | 0.031 | 0.005 | |
| D6 | 0.008 | 0.0023 | 0.14 |

**Table S3** Comparison of the defect densities at the crossover scale for MDCK-II WT and E-cad KO cells on glass. Cells were classified into groups D1-D6 according to their cell-cell distance. Statistical test: Dunn's test of multiple comparisons after a significant Kruskal–Wallis test. p-value $> 0.05$, difference is not significantly different. Source data are provided as Source Data file.

| WT | nematic | | | | hexatic | | | |
|---|---|---|---|---|---|---|---|---|
| p-value | D1 | D2 | D3 | D4 | D1 | D2 | D3 | D4 |
| D2 | 0.50 | | | | 0.23 | | | |
| D3 | 0.17 | 0.07 | | | 0.0037 | 0.0016 | | |
| D4 | 0.10 | 0.028 | 0.23 | | 0.0001 | <0.0001 | 0.008 | |
| D5 | 0.0024 | 0.0005 | 0.0032 | 0.010 | 0.0007 | 0.0010 | 0.041 | 0.26 |

| E-cad KO | nematic | | | hexatic | | |
|---|---|---|---|---|---|---|
| p-value | D3 | D4 | D5 | D3 | D4 | D5 |
| D4 | 0.20 | | | 0.27 | | |
| D5 | 0.33 | 0.24 | | 0.22 | 0.34 | |
| D6 | 0.39 | 0.23 | 0.43 | 0.39 | 0.35 | 0.27 |

**Table S4** Comparison of the shape index for MDCK-II WT and E-cad KO cells on glass, 25 kPa and 49 kPa. Cells were classified into groups D1-D6 according to their cell-cell distance. Statistical test: Dunn's test of multiple comparisons after a significant Kruskal–Wallis test. p-value $> 0.05$, difference is not significantly different. Source data are provided as Source Data file.

| | | WT | | E-cad KO | |
|---|---|---|---|---|---|
| p-value | | glass | 25 kPa | glass | 25 kPa |
| 25 kPa | D1 | 0.42 | | | |
| | D2 | 0.012 | | | |
| | D3 | 0.007 | | 0.28 | |
| | D4 | 0.012 | | 0.19 | |
| | D5 | 0.10 | | | |
| 49 kPa | D1 | 0.49 | 0.43 | | |
| | D2 | 0.07 | 0.18 | | 0.005 |
| | D3 | 0.0015 | 0.26 | 0.13 | 0.29 |
| | D4 | 0.0001 | 0.26 | 0.31 | 0.38 |
| | D5 | 0.14 | 0.37 | | |

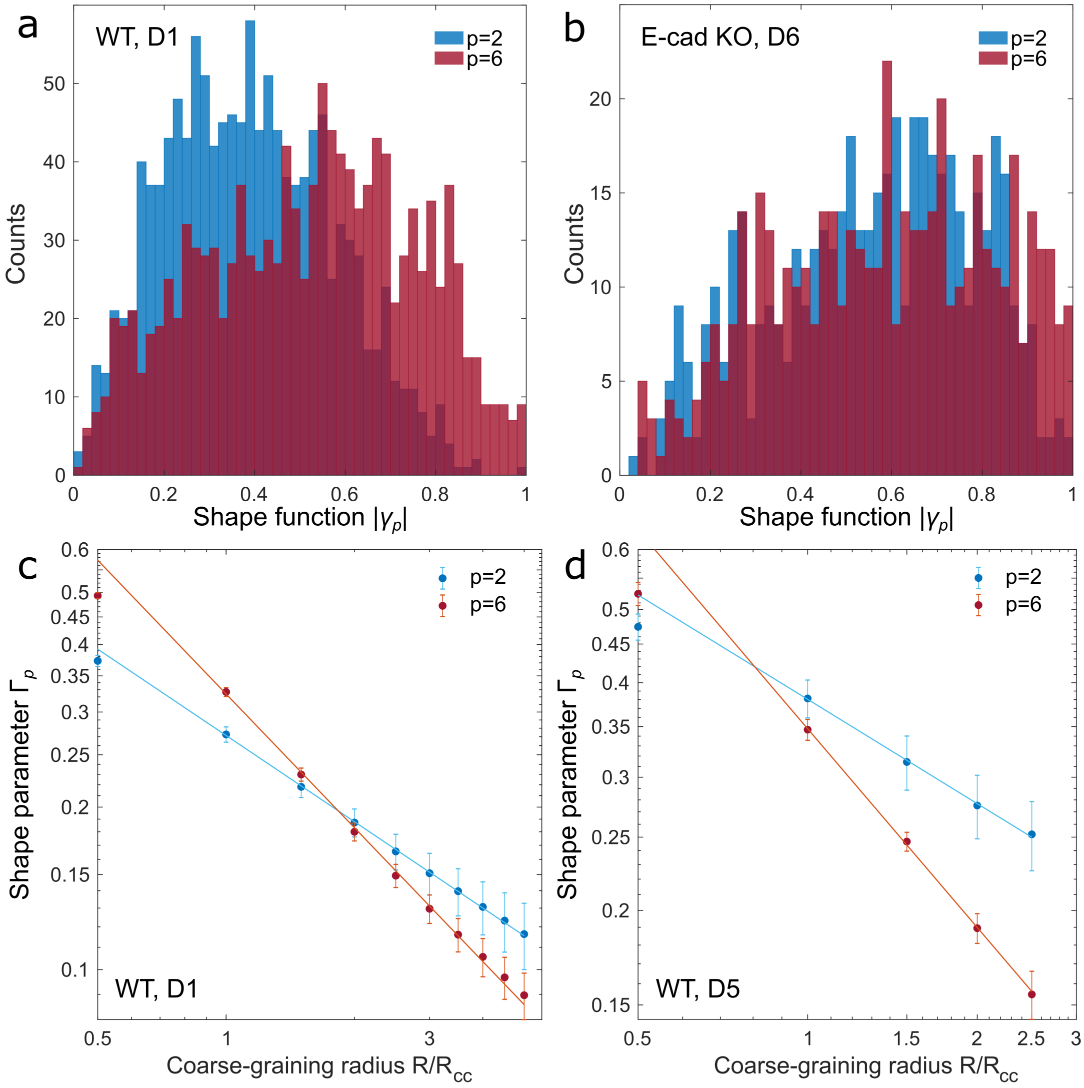


**Fig. S1 Nematic and hexatic order depend on the cell line and monolayer density. a,b**, The magnitude of the cell's shape functions of all analyzed cells within a defined density interval, as shown in Fig.2d. **a**, For MDCK-II WT cells at a high density, D1, the distribution of the hexatic shape function, $\langle|\gamma_6|\rangle$, is biased towards larger values compared to the nematic shape function, $\langle|\gamma_2|\rangle$, indicating a prevalence of hexatic order at small scale. **b**, Conversely, at low monolayer densities, D6, the two distributions overlap. **c,d**, Scale-dependent nematic and hexatic shape parameters, $\Gamma_2$ and $\Gamma_6$, of MDCK-II WT cells associated with the same density intervals D1 and D5, respectively. $\Gamma_2$ and $\Gamma_6$ decrease as power laws with the coarse-graining radius, $R/R_{\rm cc}$: $\Gamma_p \sim (R/R_{\rm cc})^{-\eta_p/2}$, with $\eta_p$ a non-universal exponent. The intersection of the fitting lines identify the hexanematic crossover scale, $R_\times$. In panel **c**, the crossover scale is $R/R_{\rm cc} = 1.83$ for cells in the highest density interval, D1, and shifts to a smaller interval at lower monolayer densities, D5, of $R/R_{\rm cc} = 0.81$ (**d**). A combined plot is shown in Fig.3f-h. Statistics: mean of $N_{\rm D1} = 12$ and $N_{\rm D5} = 8$ analysed images over 381 and 113 coarse-graining radii, respectively. Error bars represent the standard error of mean. Source data are provided as Source Data file.

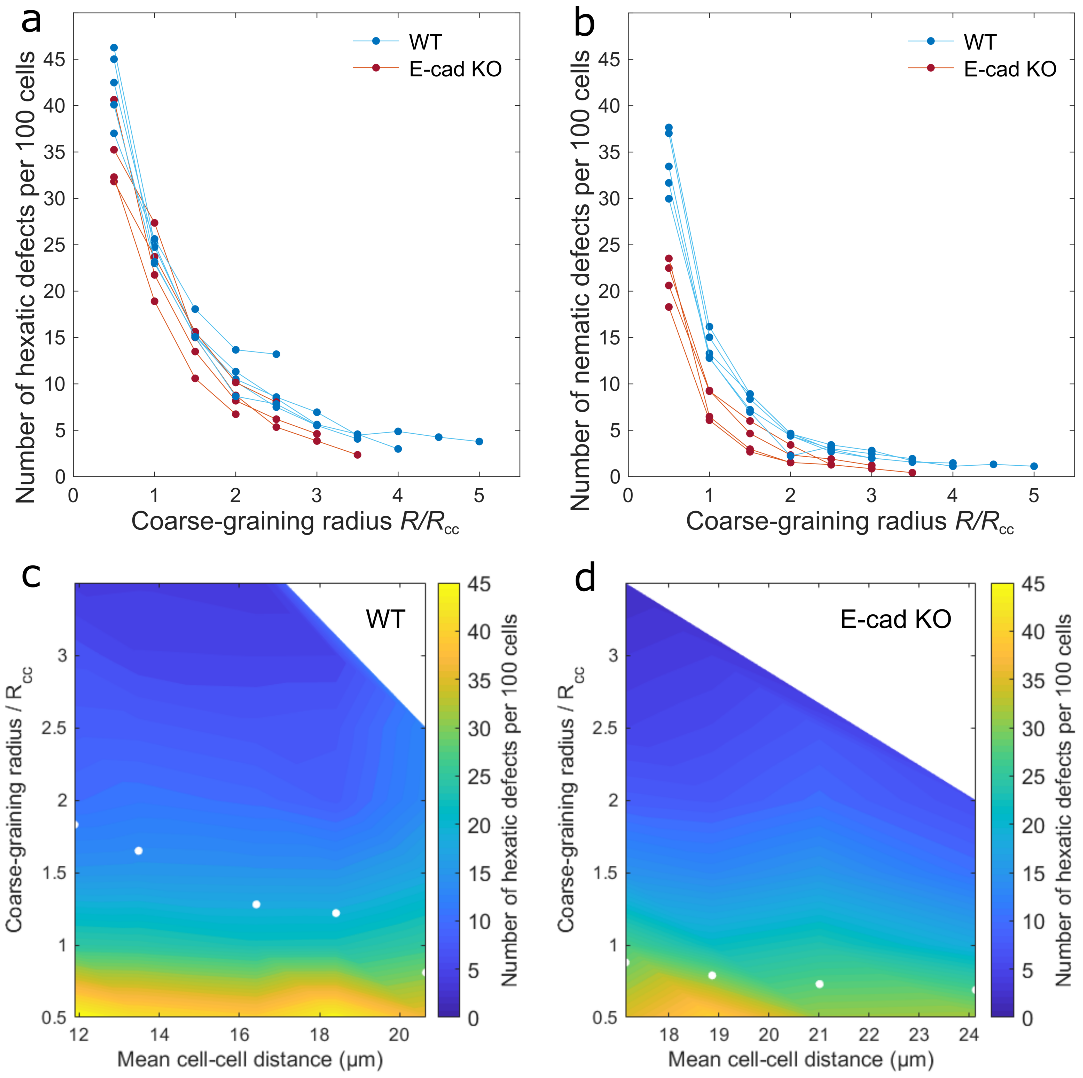


**Fig. S2 The defect density depends on the coarse-grained orientation field. a,b**, The number of nematic and hexatic defects per 100 cells decreases with increasing coarse-graining radius for MDCK-II WT and E-cad KO cells. **c,d**, The number of hexatic defects per 100 cells as a function of the coarse-graining radius and the mean cell-cell distance for both MDCK-II cell lines. Source data are provided as Source Data file.

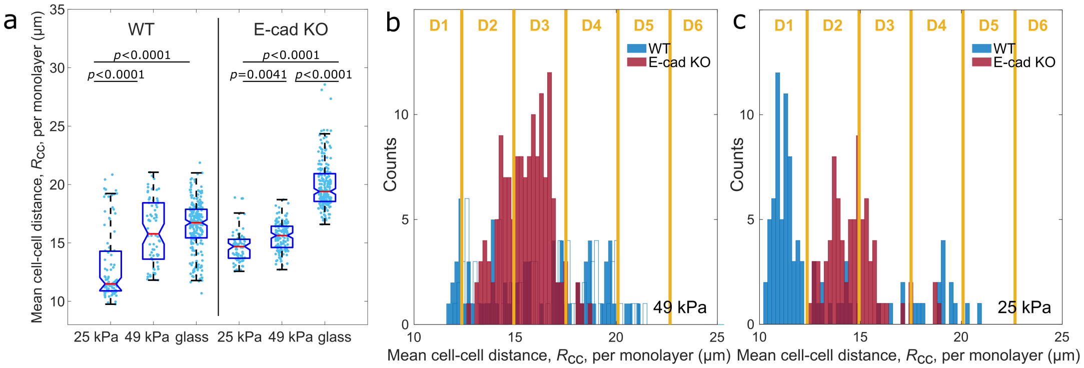


**Fig. S3 The mean cell-cell distance increases with increasing substrate stiffness.** **a**, Mean cell-cell distance of nearest neighbors for MDCK-II WT and E-cad KO cells cultured on non-coated glass, and PAA gels with a stiffness of 25 kPa and 49 kPa. Cells tend to be more compact on softer substrates. The box shows the median (red line), 25th and 75th percentiles (box), maximum and minimum without outliers (whiskers), and 95% confidence interval of the median (notches). P-values were calculated from Dunn's test of multiple comparisons after a significant Kruskal–Wallis test. **b,c**, Distributions of mean cell-cell distance of both cell lines cultured on 49 kPa and 25 kPa, respectively. Each bin-width is equal to 0.2 µm. For density dependent measurements, cells were grouped in six intervals, D1-D6, of 2.6 µm each. **a-c**, 49 kPa substrate: $N_{\mathrm{WT}} = 75$ and $N_{\mathrm{KO}} = 144$; 25 kPa substrate: $N_{\mathrm{WT}} = 95$ and $N_{\mathrm{KO}} = 81$; glass: $N_{\mathrm{WT}} = 226$ and $N_{\mathrm{KO}} = 216$ from three to five independent experiments. Source data are provided as Source Data file.
